# Minimally invasive scoliosis surgery: an innovative technique in patients with adolescent idiopathic scoliosis

**DOI:** 10.1186/1748-7161-6-16

**Published:** 2011-08-11

**Authors:** Vishal Sarwahi, Adam L Wollowick, Etan P Sugarman, Jonathan J Horn, Melanie Gambassi, Terry D Amaral

**Affiliations:** 1 Department of Orthopaedic Surgery, Montefiore Medical Center, Albert Einstein College of Medicine, Bronx, NY, USA

## Abstract

Minimally invasive spine surgery is becoming more common in the treatment of adult lumbar degenerative disorders. Minimally invasive techniques have been utilized for multilevel pathology, including adult lumbar degenerative scoliosis. The next logical step is to apply minimally invasive surgical techniques to the treatment of adolescent idiopathic scoliosis (AIS). However, there are significant technical challenges of performing minimally invasive surgery on this patient population. For more than two years, we have been utilizing minimally invasive spine surgery techniques in patients with adolescent idiopathic scoliosis. We have developed the present technique to allow for utilization of all standard reduction maneuvers through three small midline skin incisions. Our technique allows easy passage of contoured rods, placement of pedicle screws without image guidance, and allows adequate facet osteotomy to enable fusion. There are multiple potential advantages of this technique, including: less blood loss, shorter hospital stay, earlier mobilization, and relatively less pain and need for pain medication. The operative time needed to complete this surgery is longer. We feel that a minimally invasive approach, although technically challenging, is a feasible option in patients with adolescent idiopathic scoliosis. Although there are multiple perceived benefits, long term data is needed before it can be recommended for routine use.

## Introduction

Minimally invasive spine surgery is becoming more common for the treatment of multilevel pathology, including adult lumbar degenerative disorders [1-3]. The next logical step is to apply minimally invasive surgical techniques to the treatment of adolescent idiopathic scoliosis (AIS). However, there are significant technical challenges of performing minimally invasive surgery on this patient population. In contrast to adult degenerative scoliosis, the curves in AIS patients are much larger (usually 45-50° or more), the number of levels instrumented are longer (7-13), the deformity exists in three planes, and the vertebral rotation can be significant. Placement of pedicle screws (14-26 screws) also increases radiation exposure for both the patient and the surgeon [4-6]. In patients with double major curves, passing a rod that is contoured in the normal sagittal profile (thoracic kyphosis and lumbar lordosis) is a challenge in and of itself.

The ultimate goal of the surgical management of AIS is to obtain an adequate fusion. In contrast to the adult population, an anterior approach is often not utilized in AIS patients, either for release or for fusion [7]. Thus, it is imperative that any surgical technique for AIS allows for adequate fusion at the facet joint. In the context of minimally invasive surgery, obtaining sufficient surface area for arthrodesis can be challenging. Bone morphogenic protein can be utilized, but is an off-label indication for this age group as well as for this type of surgery.

Two other important issues in considering minimally invasive approaches to AIS are the length and type of skin incision as well as the reduction maneuvers employed for deformity correction. The standard stab incision for placement of minimally invasive or percutaneous pedicle screws cannot be utilized in adolescent patients, as fourteen to twenty six stab incisions in the back can be quite disconcerting for a young patient. Additionally, surgeons treating large spinal deformities typically have the ability to utilize multiple reduction maneuvers, including rod translation, rod derotation, in situ bending, direct vertebral rotation, and spine translation [8,9]. Only limited reduction maneuvers can be carried out with the present minimally invasive spine surgery instrumentation systems [10]. These instrumentation systems fall short in their ability to reduce a contoured rod into the pedicle screw heads. This is especially true when attempting to restore normal thoracic kyphosis, as a contoured rod often sticks above the screw head.

The purpose of this study is to detail a new technique for minimally invasive posterior spinal fusion which limits incision length as well as soft-tissue dissection while allowing for deformity correction.

## Materials & methods

Since May, 2008, we have been utilizing minimally invasive spine surgery techniques in patients with adolescent idiopathic scoliosis. The presented technique allows for utilization of all standard reduction maneuvers through three small midline skin incisions. This technique allows easy passage of contoured rods, placement of pedicle screws without image guidance, and adequate facet osteotomy to enable fusion.

### Surgical Technique

**Figure 1:**
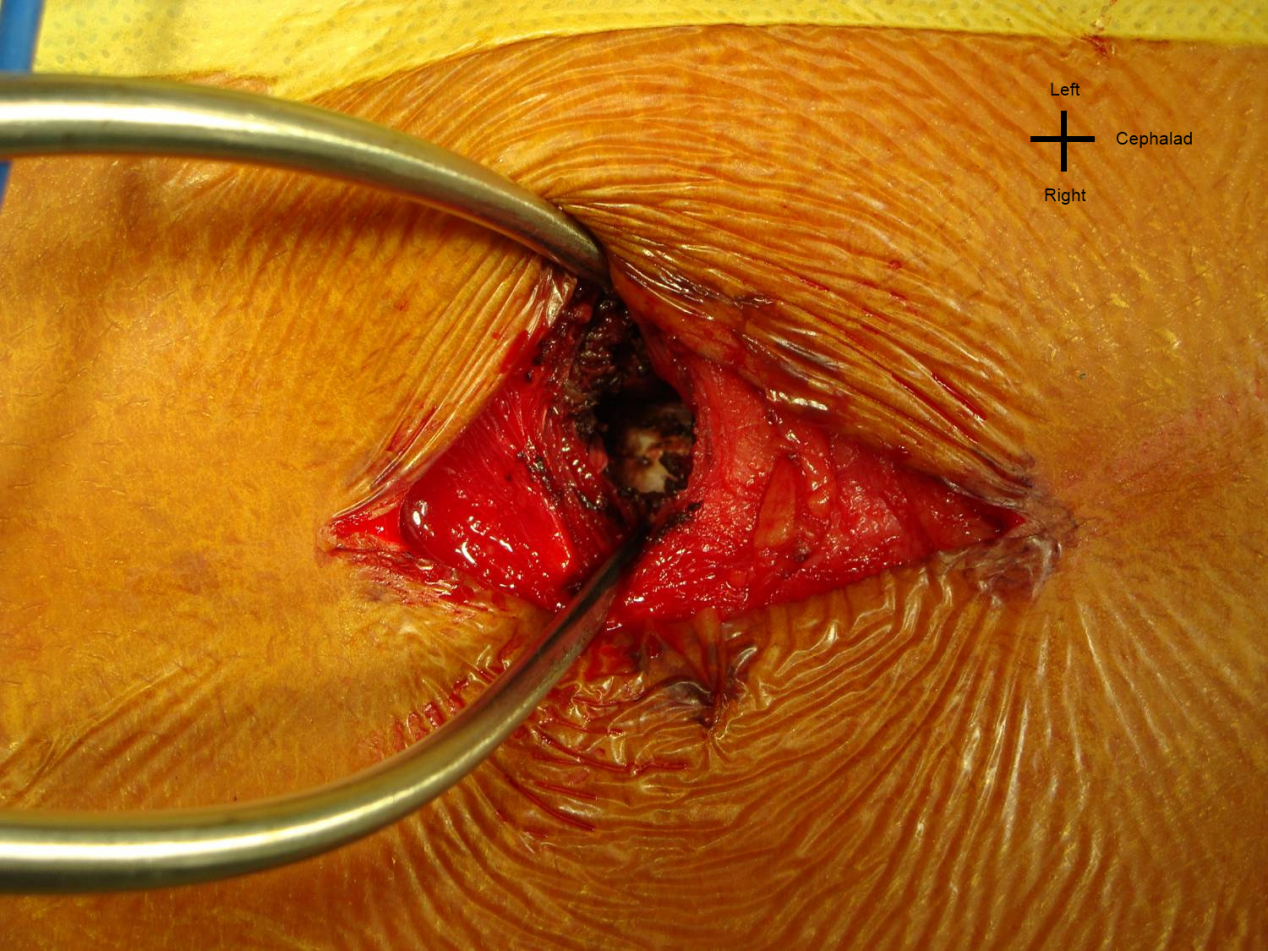
**"The L2 facet visualized through a stab incision in the fascia**." The left L2 facet visualized through a stab incision in the fascia. The Gelpi is serving as a retractor.

Three two-inch-long midline skin incisions are made for instrumentation of eleven to thirteen segments. Three to four segments (6-8 pedicle screws) are instrumented per skin incision. Intraoperative fluoroscopy is used to determine the length and location of these incisions. Although a single straight midline incision can be made, three smaller incisions are often more cosmetically appealing to patients. The skin is undermined to either side of midline in order to allow for the placement of bilateral pedicle screws. In the lumbar spine the facet can be manually palpated. A stab incision in the fascia is made directly over the facet. Thereafter, the muscle fibers are split bluntly in line with their fibers using a small cobb elevator, or with an insulated tipped electrocautery, to expose the joint (Figure 1). A small Gelpi retractor provides excellent exposure and a fiber optic light source is used for illumination. The Gelpi retractor also allows for adjustment of tissue tension, which decreases muscle necrosis and spasm. Once the facet joint is exposed, facetectomy is performed with a high speed burr. Stab incisions are made for placement of pedicle screws at all levels. At times, the stab wounds become contiguous, rendering the fascial and the muscle exposure akin to a Wiltse approach [11].

**Figure 2:**
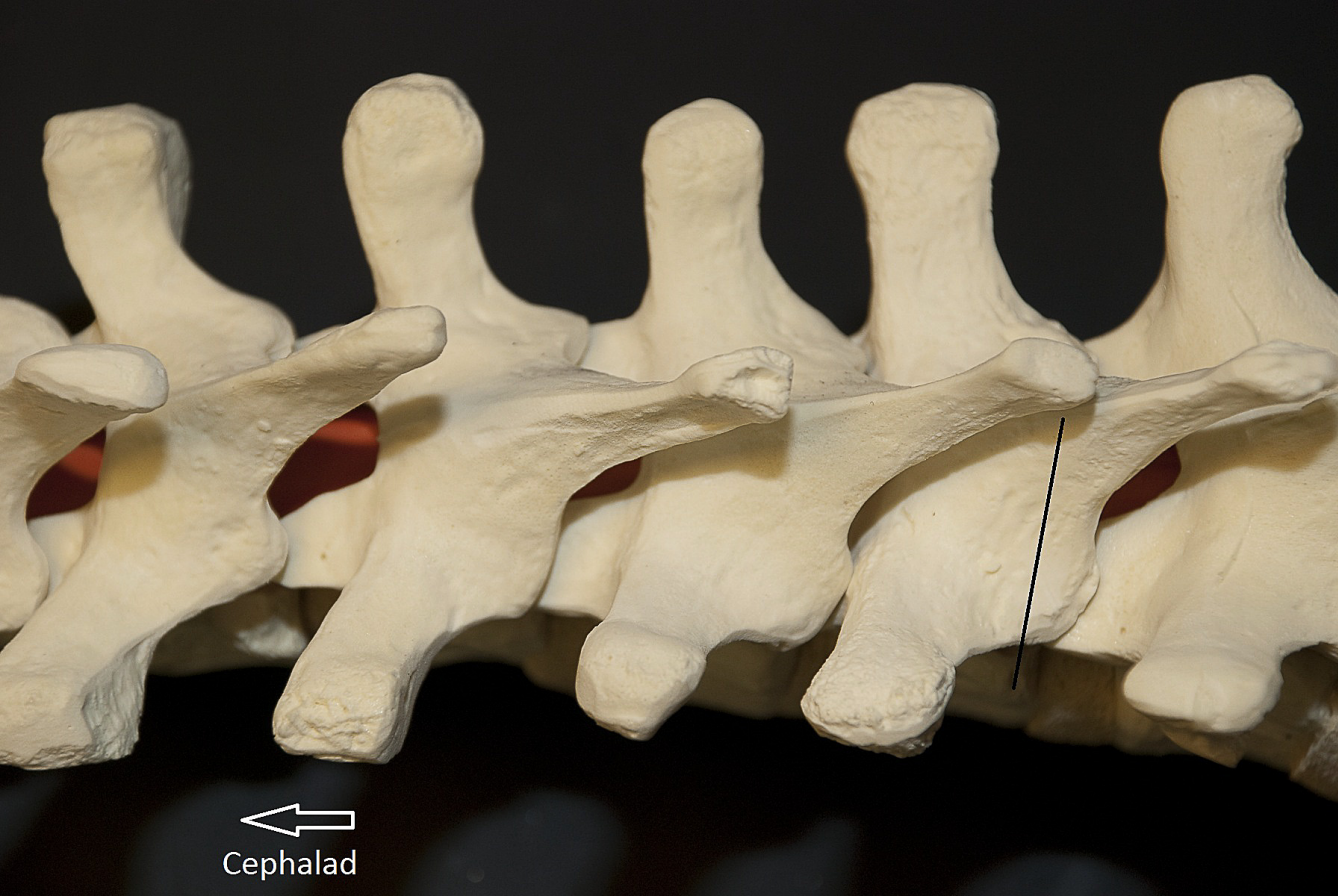
**"Localization of the facet joint**." Localization of the facet joint is as follows. The spinous process of the thoracic spine usually lies at the level of the caudal facet joint. Thus, a T6 spinous process is approximately at the level of the T7-T8 facet joint. This helps in localization of the thoracic facet. Also, notice the overhang which allows for easy dissection of the facet joint above, and the facet joint below.

In the thoracic spine the inferior facet usually lies at the level of the tip of the spinous process of the superior vertebra (Figure 2). On the concave side, we make a stab incision approximately one centimeter lateral to the midline at the level of the spinous process tip, whereas on the convex side we make a stab incision approximately 1.5 cm lateral to the midline. When in doubt, we use C-arm to confirm the location of the pedicle. We take advantage of the thoracic spine anatomy in locating the facet joints at the level above and at the level below - the overlapping laminae allow for contiguous exposure of these facets. Under direct visualization and illumination, the facet joint is osteotomized using a combination of a quarter-inch osteotome and a high-speed burr. Adequate excision of the facet joint is carried out to ensure a solid fusion. The facet joint of the uninstrumented intervening segment between skin incisions is also exposed in the manner previously described. The facet joint is osteotomized and prepared for fusion. This area is left uninstrumented, but bone graft is placed to facilitate arthrodesis. Exposure of this joint can usually be accomplished by undermining the skin and extending the fascial incision.

**Figure 3:**
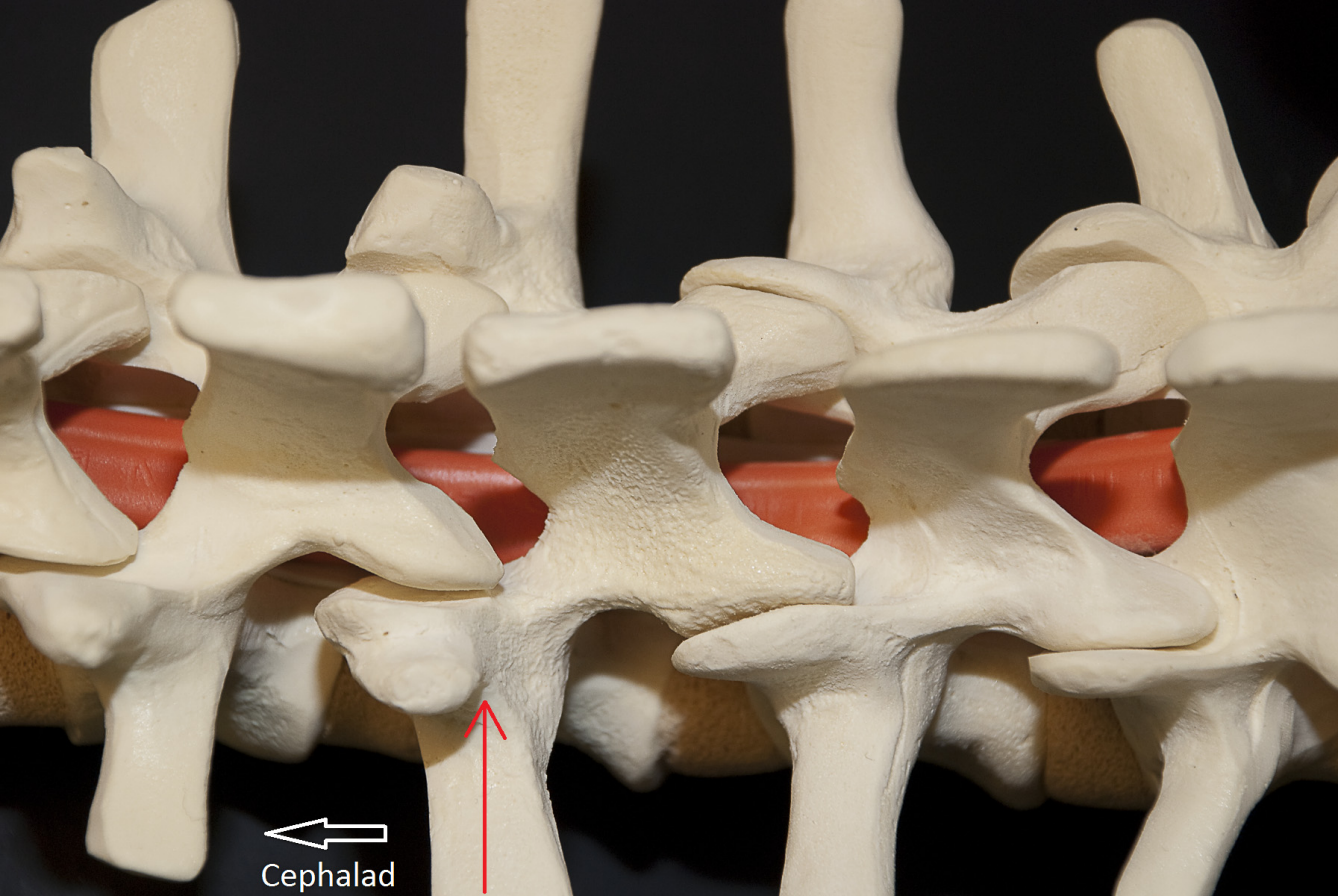
**"The entry point to the pedicle**." In the lumbar spine, the base of the superior facet often overlies the entry point to the pedicle of the same level. The superior facet can be rongeured off to expose the bleeding cancellous bony area, which marks the entry site to the pedicle.

Freehand anatomic placement technique is utilized for pedicle screw insertion, as has previously been described [12]. The facet osteotomy and the exposure allow for easy identification of the anatomic entry point. The entry point in the thoracic spine is usually the point of intersection between the midline of the facet joint and the upper-third of the transverse process. In the lumbar spine the entry is the intersection point between the midline of the transverse process and the midline of the facet joint (Figure 3). This is often at the base of the superior facet. Complete exposure of the transverse process is usually not needed for identification of this landmark, but may be carried out if desired. Alternatively, the pedicle screw can be placed using fluoroscopy guidance. We prefer to place all screws on one side first. This allows for easy mobilization of the skin. It is typically not possible to place both screws in each vertebra simultaneously due to the limited skin incision.

**Figure 4:**
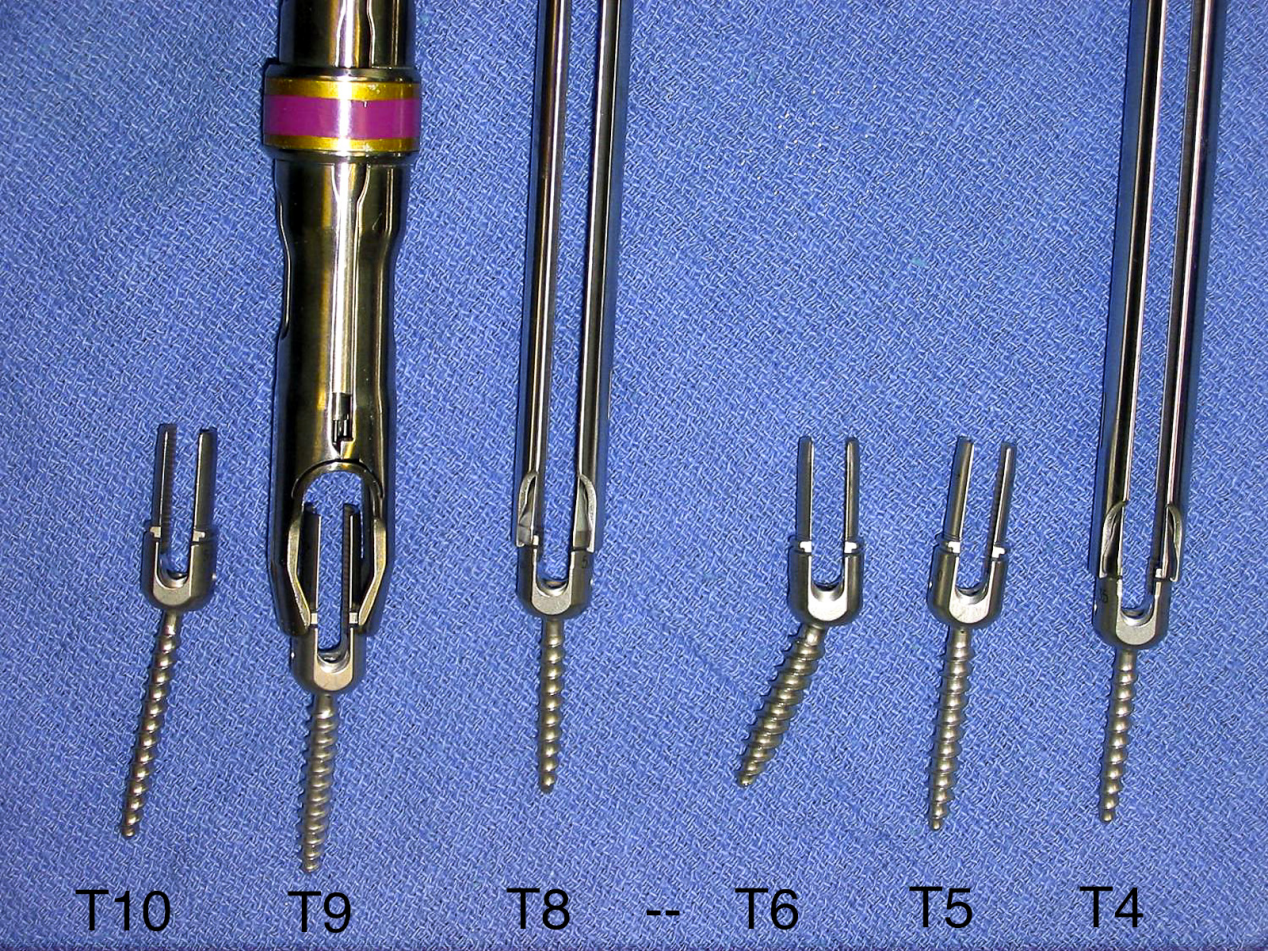
**"Order of screw placement**." The order of screws placement for two segments (7 Spinal levels) is shown from left to right representing inferior to superior. Each segment consists of three levels with a skipped level in between each segment. Two standard reduction screws (T10, T9) are instrumented at the inferior levels followed by a MIS reduction screw (T8) at the superior level. The same pattern is seen in the T4-T6 segment. A rod reduction device is shown on the T9 screw which can facilitate seating of the rod when necessary. Open connectors are pictured extending from the MIS screws.

We utilize the DePuy 5.5 EXPEDIUM^® ^(5.5 mm rod, stainless steel) pedicle screw instrumentation system. Our preference is to use two uniaxial reduction screws and one MIS screw with an open connector per skin incision (Figure 4). The reduction screws have significant advantages. They allow for easy reduction of the rod and/or the spine to achieve correction of scoliosis deformity in two planes. Since the extended tabs stick out above the fascia, they allow for easy passage of the rod into the screw heads. Thus, the rod can be passed under direct visualization and the screw heads can be manipulated for easier passage. The extended tabs also serve as soft tissue retractors. Prior to the insertion of the pedicle screw, the fusion site is prepared. Local autograft (from the facet joint osteotomy) as well as a small BMP-enriched sponge or other bone graft substitute is placed in the fusion bed. We usually do not perform EMG stimulation of the pedicle screws, but SSEP and transcranial MEP monitoring are employed throughout the case.

**Figure 5:**
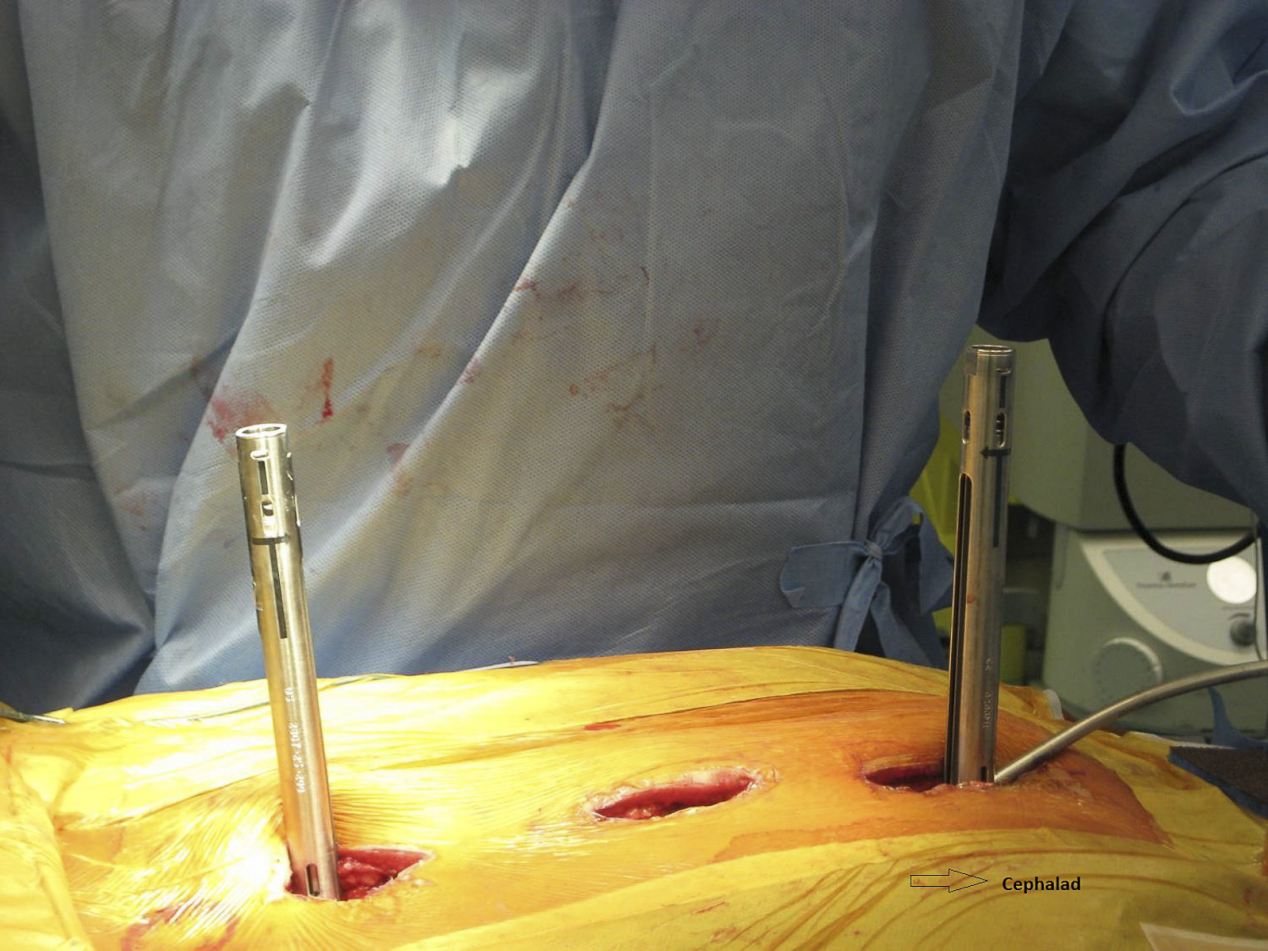
**"Rod introduction from cephalad to caudad**." The rod is being introduced cephalad to caudad. Notice the open-ended MIS connector at the proximal and distal incisions. These serve as posts against which the rod can be manipulated and also enable easy visualization of the rod.

**Figure 6:**
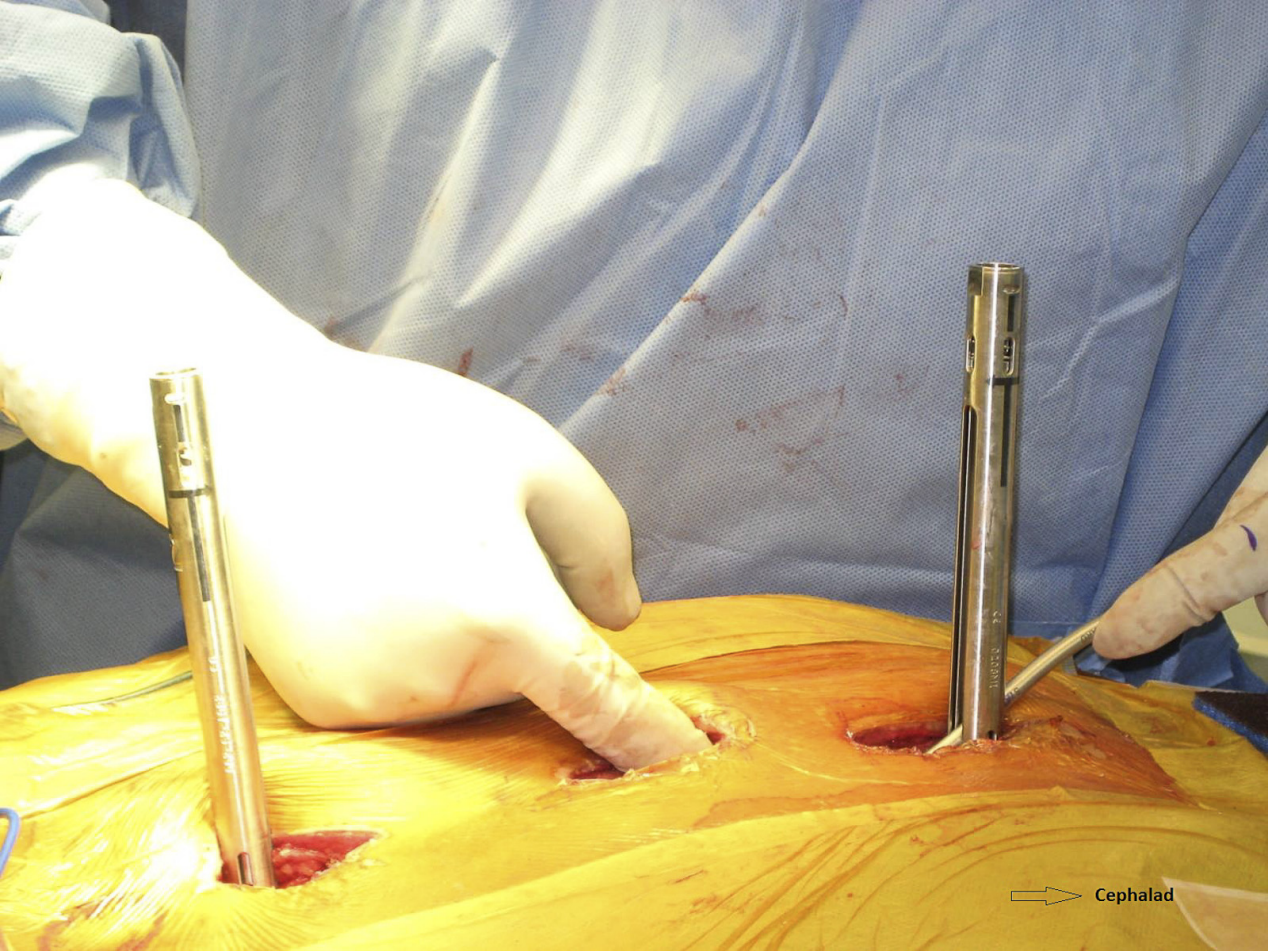
**"Passing the rod along the spine**." As the rod is passed along the spine, it can be manually palpated, as show in the picture, and guided as necessary. Notice the kyphotic bend of the rod which is being passed to maintain the desired sagittal profile.

Two rods, cut to appropriate length, are contoured in the normal sagittal plane to reproduce desired thoracic kyphosis and lumbar lordosis. The rod is inserted cephalad to caudad (Figure 5). This is an important safety step, as the overlapping laminae in the thoracic spine prevent an inadvertent entry into the spinal canal. The reduction screws are capped as the rod advances, and the screw heads can be manipulated for rod passage. The open MIS connectors serve as a post against which the rod can be maneuvered. A vise-grip pliers is utilized to hold the rod as it provides a strong grip and easy manipulation. A rod pusher can be utilized to translate the rod onto the screw heads. Alternatively, the assistant can directly apply translation force to the chest wall to push the spine over to the rod. We prefer to utilize rod translation maneuvers, however, a CD rod derotation technique can also be utilized with the help of a vise-grip. As the rod passes from one wound to the next, one must confirm that the rod is lying beneath the fascia. The rod can be manually palpated in the distal wound (Figure 6), which helps to properly direct the rod.

**Figure 7:**
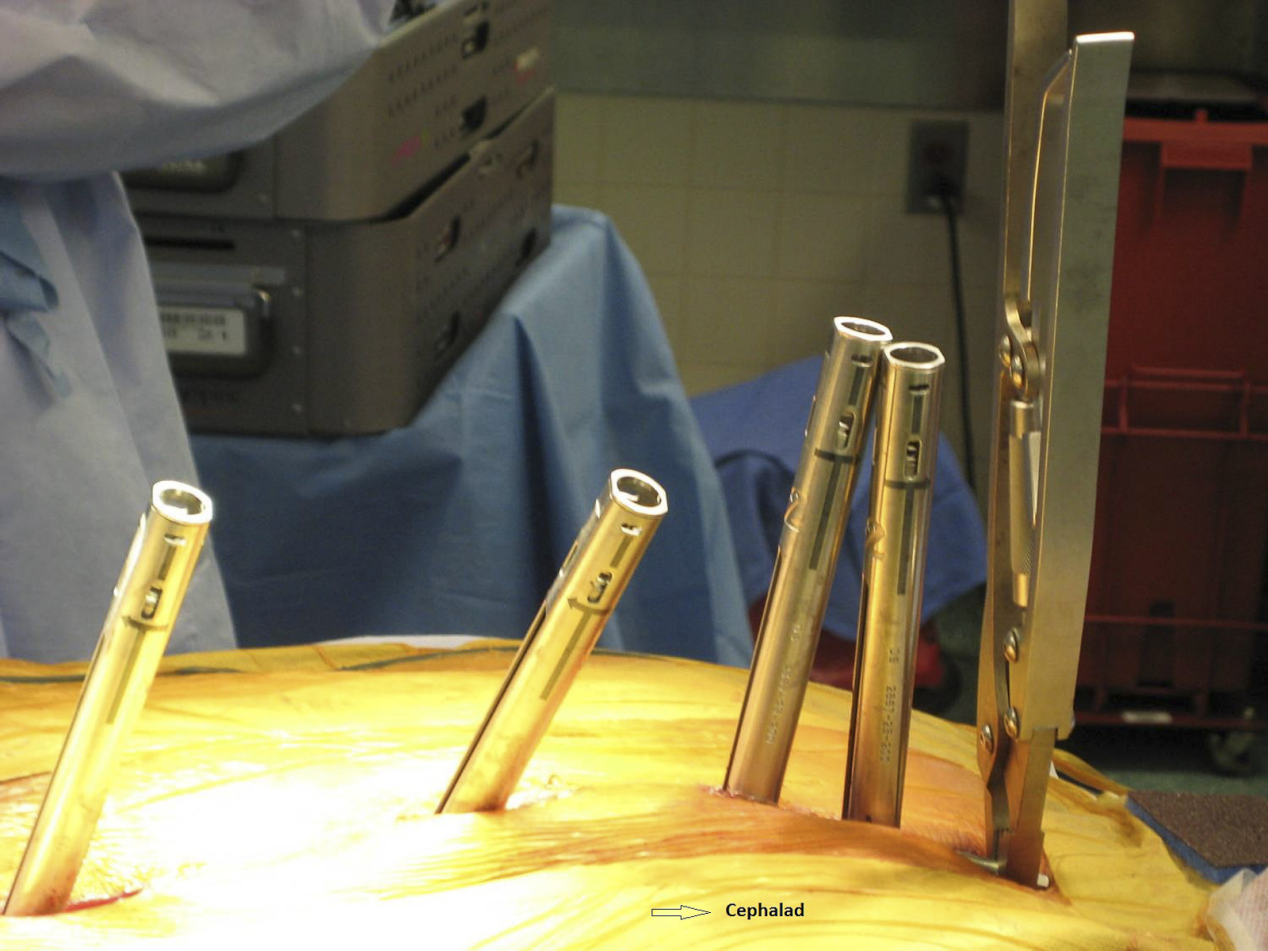
**"Rod manipulation with a vise-grip for larger deformity**." The vise-grip allows a stronger manipulation of the rod, which is often needed in larger deformities. It can also allow for a CD rod-derotation maneuver. Notice that the rod has almost completely disappeared from the picture. Usually the rod is longer than needed, and at this point, can be cut to appropriate length. Also notice the sagittal contour of the rod has been appropriately maintained. The extra open MIS connectors used here are an occasional variation in technique.

**Figure 8:**
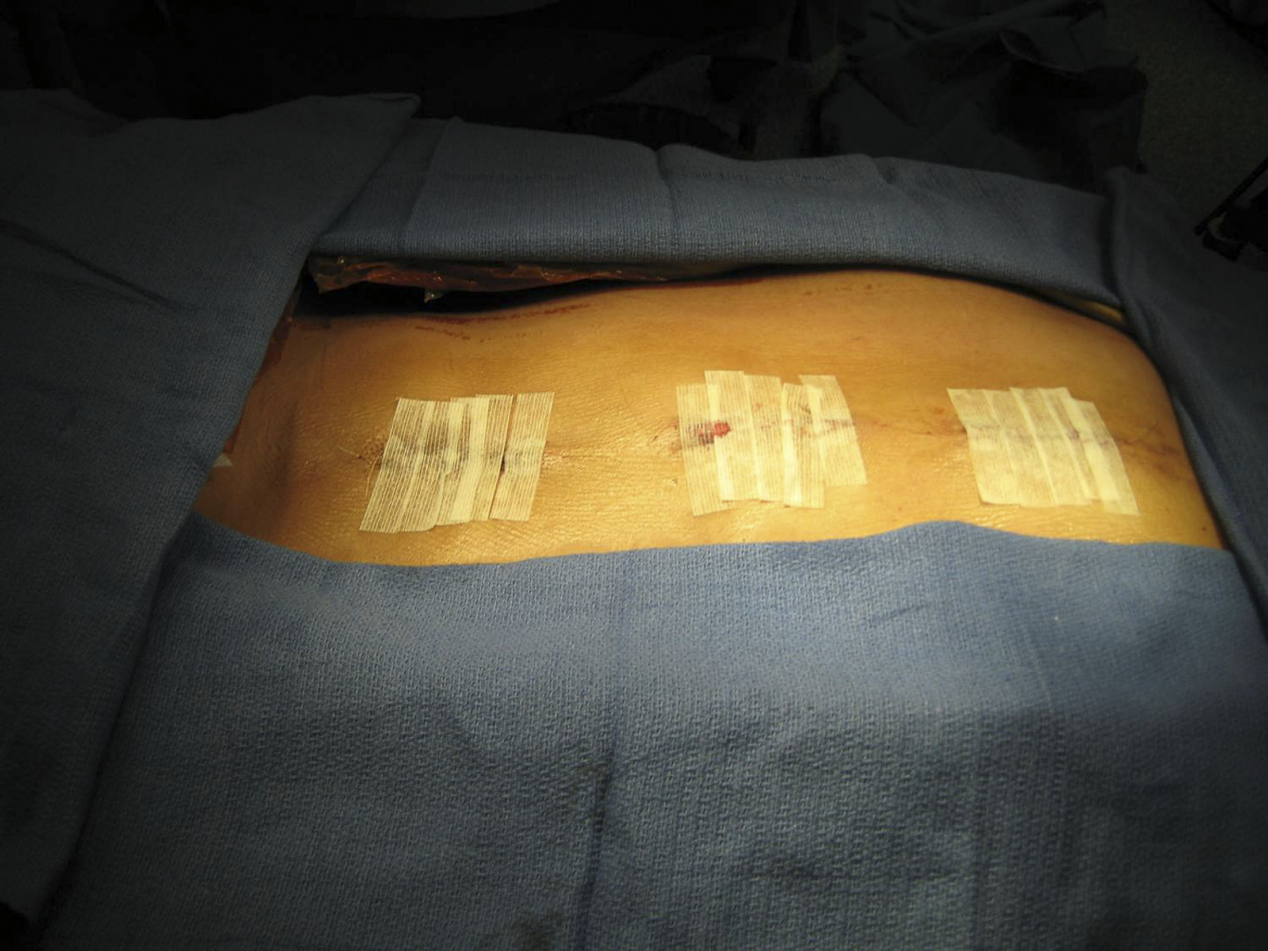
**"The wound after skin closure**." The wound after skin closure. The wound, as a standard, is closed in layers. We now prefer to excise the wound edges prior to closure to prevent hypertrophic scar formation.

Once the rod is seated in all pedicle screw heads, the rod is manipulated to restore the sagittal contour of the spine (Figure 7). Caution must be given to prevent over-rotation of the rod, as this has the potential to reverse the thoracic kyphosis. This mistake is more likely to occur with a rod derotation maneuver, as the sagittal contours of the rods are difficult to appreciate through small skin incisions. The set screws are now sequentially tightened, and the rod is formally seated. The same maneuver is now carried out on the opposite side following the placement of pedicle screws. Appropriate compression and distraction force can also be carried out. This can be easily accomplished through the midline skin incisions. In situ rod bending can also be carried out, but is not our preferred technique. A direct vertebral rotation maneuver is then carried out off the concave-side screws. Triangulation technique is difficult to achieve, as the skin incision limits access to both sides simultaneously. This can, however, be easily carried out with a straight midline incision approach. Closure is fairly rapid, and is carried out in a layered fashion. We do not utilize drains. In our more recent cases, we excise the wound edges to prevent hypertrophic scar formation (Figure 8). While the patient is still on the operating table, anteroposterior and lateral X-rays are taken to confirm adequate correction in both planes. Our postoperative protocol is fairly routine. We do not routinely use a brace. Patients must be able to walk up a flight of stairs prior to hospital discharge, and are to remain out of school for six weeks to three months. At two weeks post-op, patients may go outside their residence, and at three weeks post-op, they may travel short distances. We allow activity as tolerated within the limits of pain. Patients can return to gym class between four and six months post-op, and may participate in contact sports and perform heavy lifting after six months. We prefer to use Ketorolac (Toradol, Roche Laboratories, Nutley, NJ) rather than morphine for analgesia purposes.

## Results

**Figure 9:**
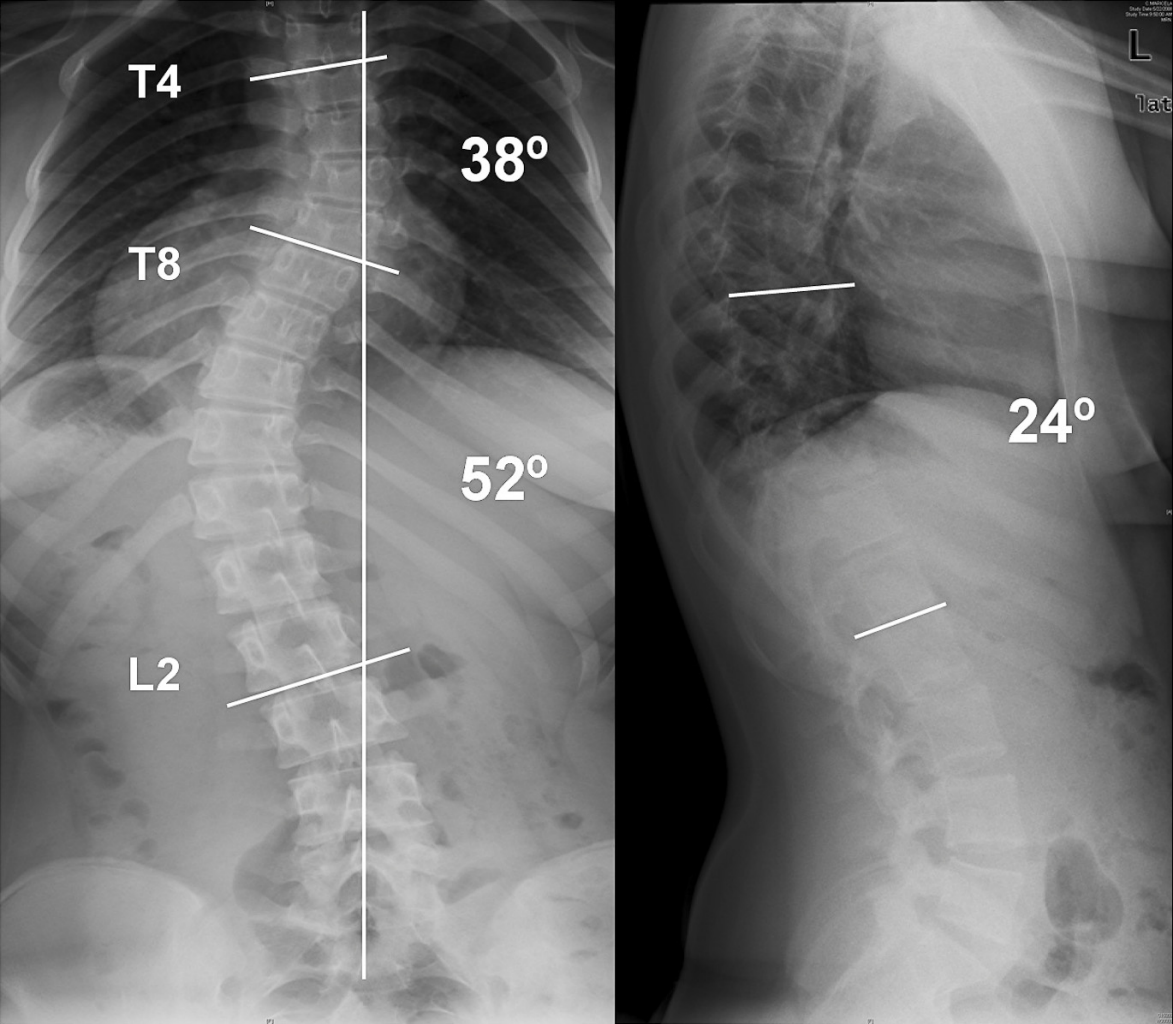
**"Patient 1 - Anteroposterior and lateral preoperative radiographs**." Patient 1 - Anteroposterior and lateral radiographs show a 52° main thoracic curve and a 38° upper thoracic curve, with 24° dorsolumbar kyphosis. MIS technique was utilized (Figure 10 & 11), and both of the curves were instrumented.

**Figure 10:**
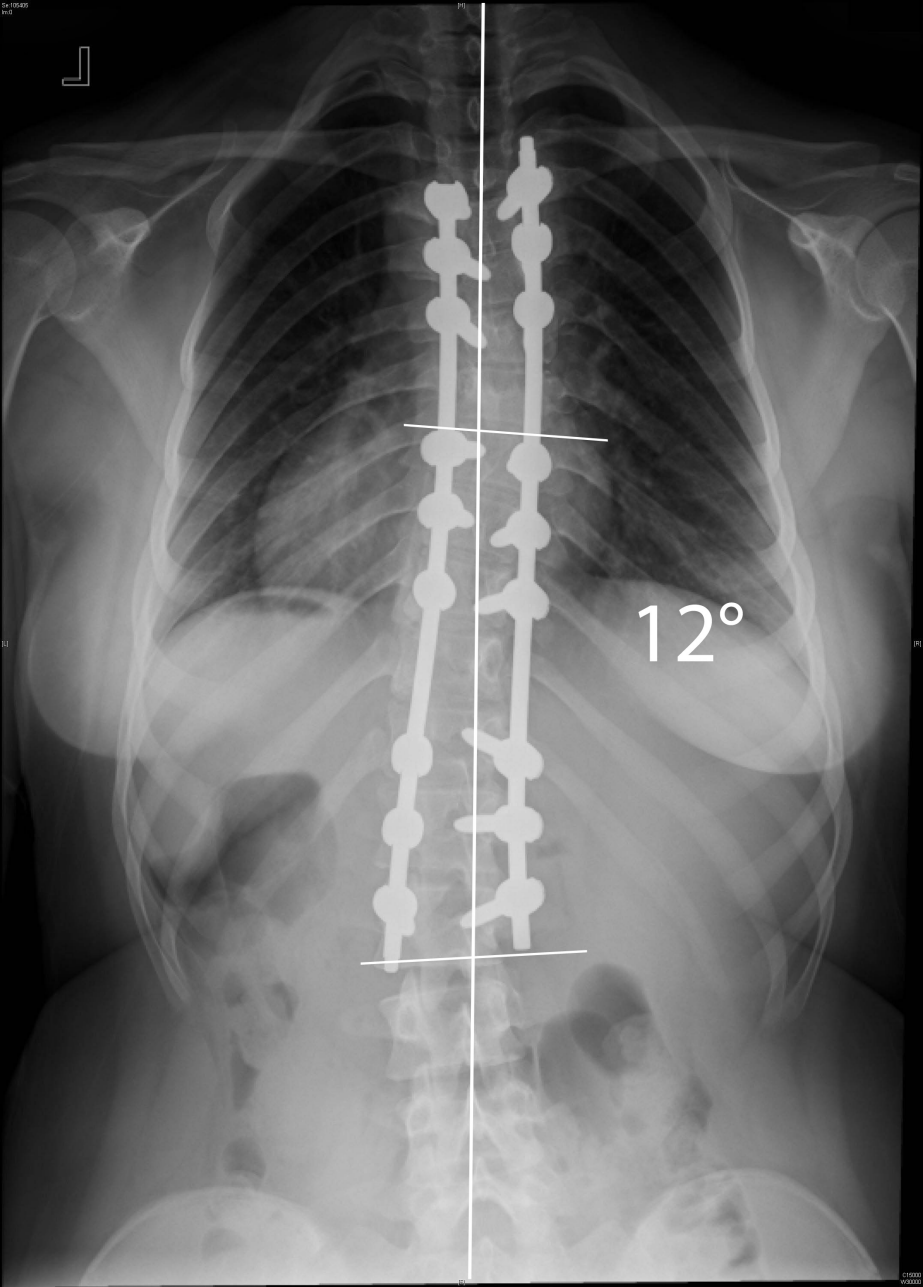
**"Patient 1 - 2-year postoperative anteroposterior and lateral radiographs**." 2-year postoperative correction anteroposterior and lateral X-rays of the patient in Figure 9. A 3-3-3 pattern of instrumentation was utilized. The patient shows good correction, is well balanced in the coronal plane, the shoulders are level, and the dorsolumbar kyphosis has been restored to normal.

**Figure 11:**
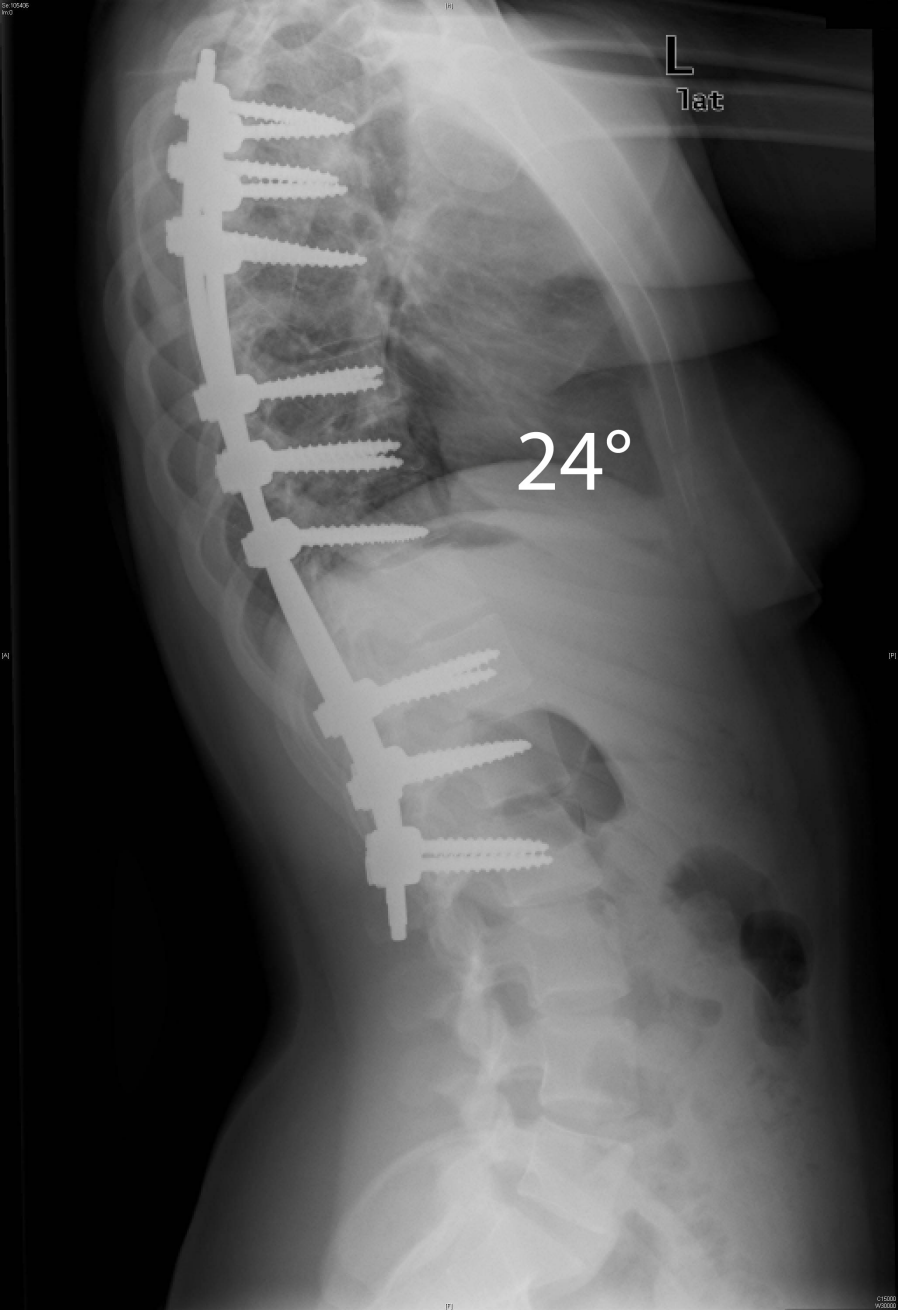
**"Patient 1 - 2-year postoperative anteroposterior and lateral radiographs**." 2-year postoperative correction anteroposterior and lateral X-rays of the patient in Figure 9. A 3-3-3 pattern of instrumentation was utilized. The patient shows good correction, is well balanced in the coronal plane, the shoulders are level, and the dorsolumbar kyphosis has been restored to normal.

**Figure 12:**
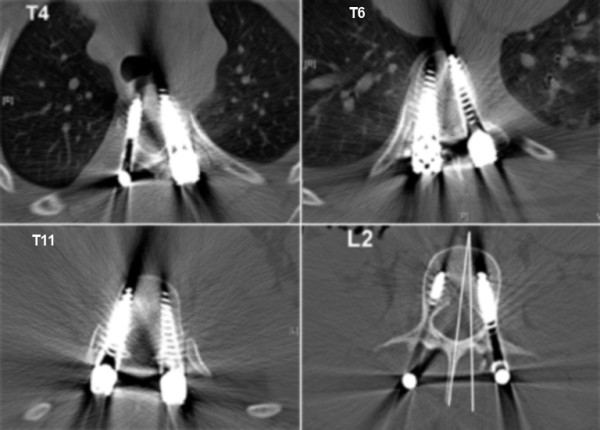
**"Patient 1 - postoperative axial CT-Scan.**" The postoperative CT-scan of the patient shows adequate placement of the pedicle screws (6-0 × 40 mm screws were commonly utilized). Also notice that the lowest instrumented vertebra (L2) has been restored to nearly neutral rotation.

Two case examples are illustrated here to show the degree of correction that can be achieved with the MIS technique. In our first case, the patient is a 14 year-old girl with a 52° left-sided main thoracic (T8-L2) and a 38° (T4-T8) upper thoracic curve. The patient can be seen to have a 24° thoracolumbar kyphosis (Figure 9). The classic 3-3-3 pedicle instrumentation pattern (three levels instrumented per skin incision) was utilized. Postoperative X-rays are shown (Figure 10 &11). 6-0 × 40 mm screws were used at all levels and were placed using a freehand anatomic technique. Postoperative CT-scan is used to evaluate adequacy of pedicle screw placement (Figure 12).

**Figure 13:**
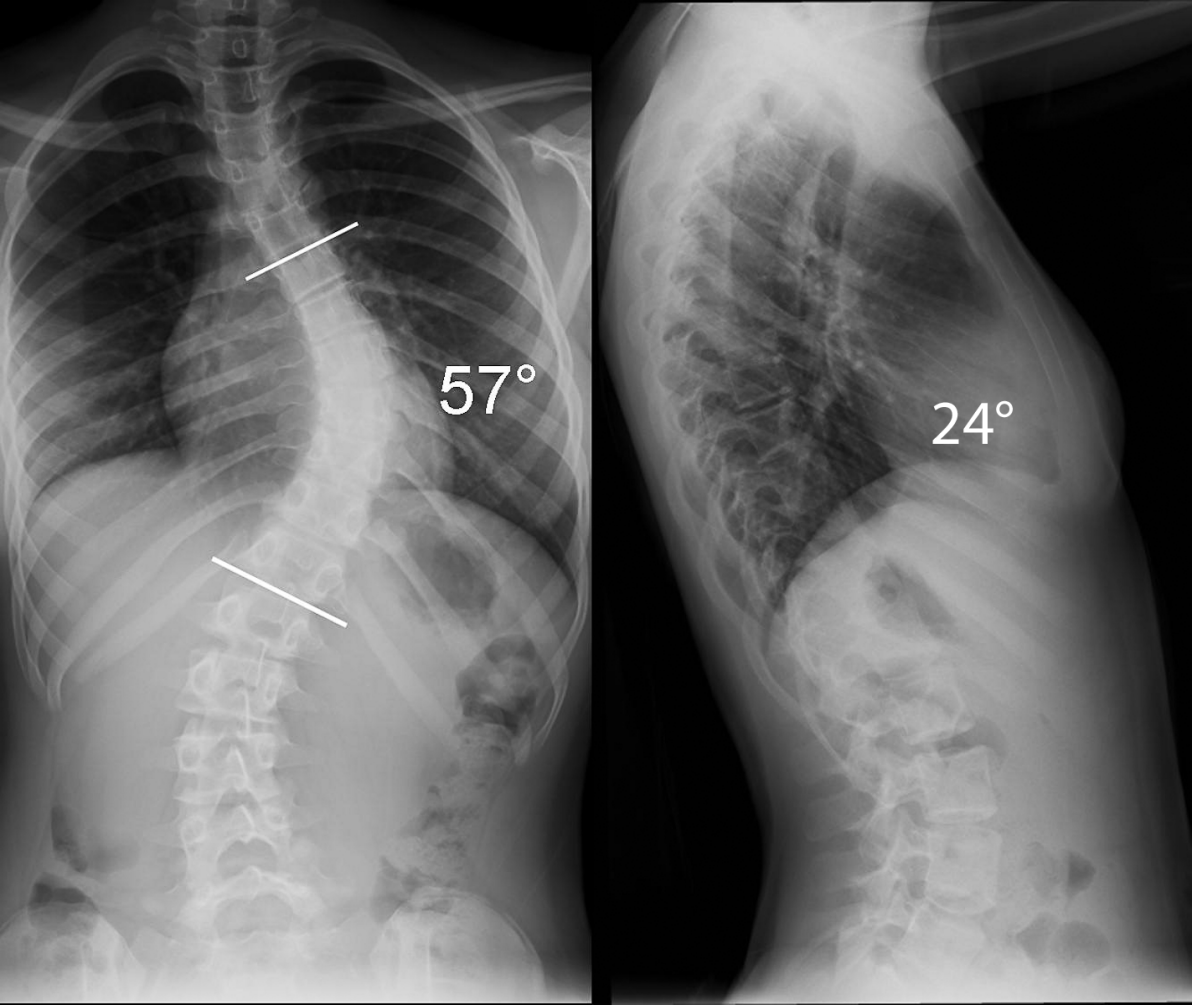
**"Patient 2 - Anteroposterior and lateral preoperative radiographs**." Anteroposterior and lateral radiographs show a 57° main thoracic curve which was corrected using the MIS technique. A slightly different pattern of pedicle screws was utilized in this patient, as shown in Figure 14 & 15.

**Figure 14:**
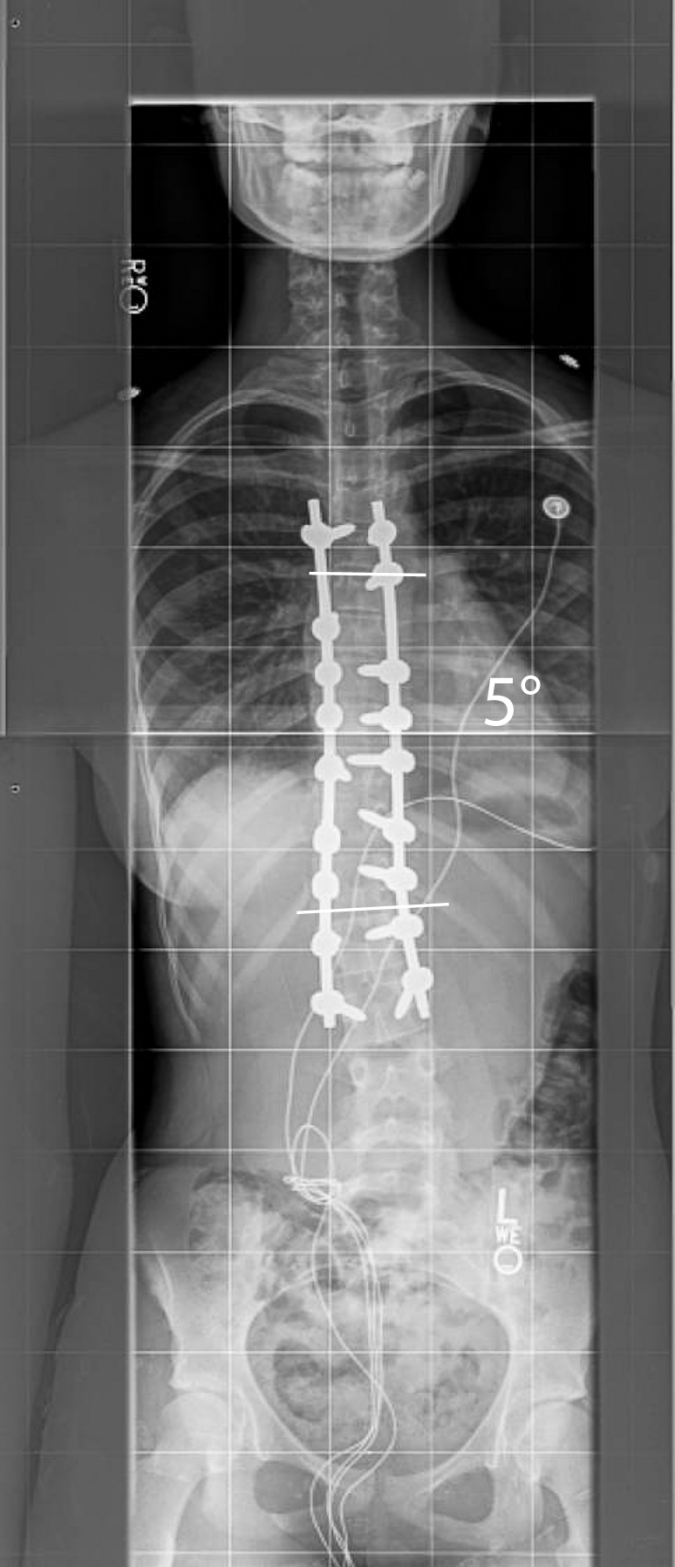
**"Patient 2 - Postoperative anteroposterior and lateral radiographs**." Postoperative images of the patient in Figure 13. A slightly different instrumentation pattern has been used (3-3-4 levels have been instrumented per incision). A good correction in both planes has been obtained, the shoulders are nearly level and the patient is balanced in the coronal plane.

**Figure 15:**
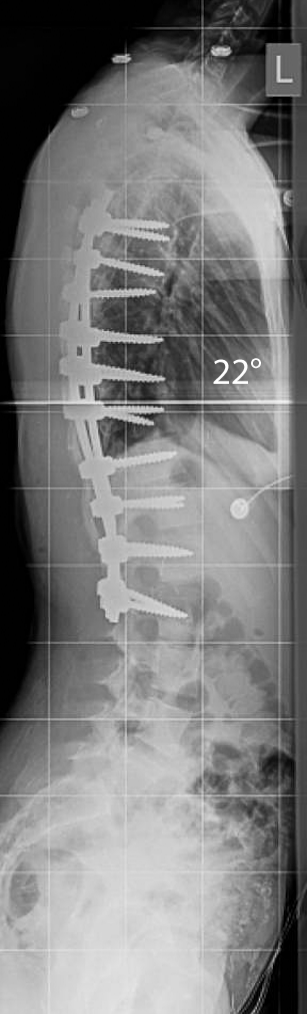
**"Patient 2 - Postoperative anteroposterior and lateral radiographs**." Postoperative images of the patient in Figure 13. A slightly different instrumentation pattern has been used (3-3-4 levels have been instrumented per incision). A good correction in both planes has been obtained, the shoulders are nearly level and the patient is balanced in the coronal plane.

In our second case a 13.5 year-old girl with a 57° right-sided main thoracic curve (T6-T12) underwent minimally invasive surgery for correction of her spinal deformity. A slightly different variation of screw placement was utilized (Figure 13). Four levels were instrumented through the distal incision, while three levels were instrumented through the proximal and middle incisions. The postoperative X-rays demonstrate this variation and show good correction in the coronal and sagittal planes (Figure 14 &15).

Two-year follow-up data for 7 patients with AIS who underwent posterior spinal fusion with our minimally invasive technique was obtained. The average age in this cohort was 15.6 (range: 12.6 - 20.5) years. 4 patients had Lenke type 1 curves, 2 had Lenke type 2 curves, and 1 had a Lenke type 5 curve. The cohort (6 female; 1 male) had a mean preoperative Cobb angle of 47.7° (range: 40° - 54°) and a mean preoperative kyphosis angle of 20.4° (range: 11° - 28°). The mean length of surgery was 8.71 ± (range: 7.25 - 9.66 hours), and average estimated blood loss was 564.3 (range: 100 - 1000 cc). Postoperative radiographic evaluation revealed a mean 8.66° (range: 4° - 11°) curve postoperatively, translating to an 81.67% (range:75.8% - 92.6%) curve correction, with good maintenance of correction over the course of follow-up. CT based evaluation showed complete facet joint arthrodeses and revealed that 90.70% of pedicle screws were accurately placed within the cortical walls. A 2-year follow-up study by our group indicates that this minimally invasive technique provides similar deformity correction as a standard open posterior spinal fusion [13].

## Discussion

**Figure 16:**
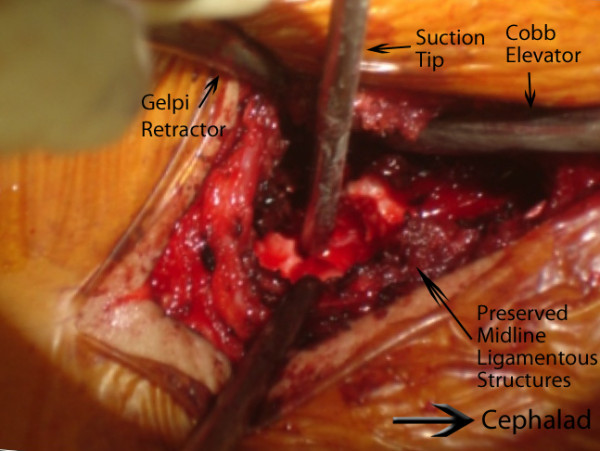
**"A trans-foraminal modified approach to a Ponte type osteotomy**." A trans-foraminal modified approach to a Ponte type osteotomy. We use this trans-foraminal osteotomy (TFO) for larger and less flexible curves. This approach allows for resection of the entire facet joint and ligamentum flavum. In the figure, notice a wide bone resection has been carried out while preserving the midline osteo-ligamentous complex. The tip of the suction is resting on the dura. In idiopathic cases, the ligamentum flavum can be left behind after resection of the facet joint.

Currently, we have limited use of this technique to curves which are less than 70° and reasonably flexible in order to limit the difficulty of these initial cases. For less flexible curves, we have utilized a transforaminal approach to a Smith-Peterson type osteotomy. This transforaminal osteotomy allows excision of the entire facet, facet joint capsule, and ligamentum flavum, while preserving the midline ligamentous and bony structures (Figure 16).

Our initial results indicate that comparable correction can be achieved to the standard pedicle screw technique both in the coronal and sagittal planes. In flexible curves, correction of 75-80% can be achieved. In addition, multiple other advantages to a minimally invasive technique include: less blood loss, shorter hospital stay, earlier mobilization, as well as less pain and need for pain medication [3,14]. However, further investigation is needed before similar claims can be made for this technique. The operative time needed to complete this surgery is longer. However, this is expected in the learning of a new technique. We have found that the scar in some patients may be, paradoxically, broader. This may be due to constant retraction of the wound edges. We now excise the wound edges prior to closure, with improved results postoperatively.

The dosage and long term effects of using BMP in patients of childbearing age have not yet been fully established. Mulconrey and Lenke et al. ("Safety and efficacy of bone morphogenetic protein [rhBMP-2] in a complex pediatric spinal deformity at a minimum 2-year follow-up." Presented at International Meeting on Advanced Spine Techniques, July 8-11, 2008, Hong Kong) assessed the safety and efficacy of BMP in 20 patients treated for complex pediatric spine deformity with a minimum 2-year follow-up. One incidence of infection was reported, but there were no other complications. Additionally, they found a 94% fusion rate over 118 levels using 5.9 mg/level of BMP-2 (INFUSE - Medtronic Sofamor Danek, Memphis, TN). In a recent review of the literature, Betz et al. concluded that BMP may be promising for enhancing fusion or as a bone graft substitute [15]. We have elected to use one large kit of BMP (12 mg total dose of INFUSE (1.5 mg/cc) - Medtronic Sofamor Danek, Memphis, TN) in these patients in order to ensure adequate fusion takes place. The long-term non-union rates in our patient population are not yet known, however, the short-term data is promising.

The efficacy of ketorolac in postoperative pain management in pediatric surgical populations has been assessed, and its benefits in adolescent spinal fusion surgery significantly outweigh its risks. Ketorolac effectively controls pain in pediatric surgery, and does not share the adverse effects of opioids, which include nausea and vomiting, respiratory depression, constipation, drowsiness, and potential for abuse [16]. Furthermore, patients treated with ketorolac have less postoperative pain resulting in a shorter hospital stay, leading to lower hospital costs overall [17]. One retrospective study examining the effect of NSAIDs in adult spinal fusion patients demonstrated that ketorolac has a significant inhibitory effect on spinal fusion [18]. However, more recent studies on the adolescent population indicate that ketorolac does not influence the development of pseudoarthrosis after posterior spinal fusion in adolescent idiopathic scoliosis and does not increase risk of reoperation in children who underwent spinal surgery [16,17]. The clinical evidence that ketorolac is superior to morphine in terms of side effects and cost suggest that it be the analgesic of choice for minimally invasive scoliosis surgery.

## Conclusions

We feel that a minimally invasive approach, although technically challenging, is a feasible option in patients with adolescent idiopathic scoliosis. Although there are multiple perceived benefits, long term data is needed before it can be recommended for routine use.

## Competing interests

The authors declare the following competing interests:

AW: Consultant/Speaker - Depuy Spine, Inc.

VS: Consultant/Speaker - Depuy Spine, Inc.

Consultant - Medtronic, Inc.

AW, VS, TA: Research Grant - Stryker Corp.

## Authors' contributions

VS was responsible for designing the instrumentation technique, data review, and draft and review of the manuscript.

AW was responsible for designing the instrumentation technique and manuscript review.

ES was responsible for data collection and review, manuscript drafting, and manuscript review.

JH was responsible for manuscript revision, and data collection.

MG was responsible for data collection and review.

TA was responsible for designing the instrumentation technique, and manuscript review.

All authors read and approved the final manuscript.
